# Effects of the Combination Treatment of Raloxifene and Alendronate on the Biomechanical Properties of Vertebral Bone

**DOI:** 10.1002/jbmr.197

**Published:** 2010-08-04

**Authors:** Tamim Diab, Jason Wang, Susan Reinwald, Robert E Guldberg, David B Burr

**Affiliations:** 1 Department of Anatomy and Cell Biology, Indiana University School of Medicine Indianapolis, IN, USA; 2 Parker H Petit Institute for Bioengineering and Bioscience, Georgia Institute of Technology Atlanta, GA, USA; 3 George W Woodruff School of Mechanical Engineering, Georgia Institute of Technology Atlanta, GA, USA; 4 Department of Biomedical Engineering, Indiana University–Purdue University at Indianapolis Indianapolis, IN, USA

**Keywords:** RALOXIFENE, ALENDRONATE, BIOMECHANICAL PROPERTIES, FRACTURE, DENSITY

## Abstract

Raloxifene (RAL) and alendronate (ALN) improve the biomechanical properties of bone by different mechanisms. The goal here was to investigate the effects of combination treatment of RAL and ALN on the biomechanical properties of vertebral bone. Six-month-old Sprague-Dawley rats (*n* = 80) were randomized into five experimental groups (sham, OVX, OVX + RAL, OVX + ALN, and OVX + RAL + ALN; *n* = 16/group). Following euthanization, structural and derived material biomechanical properties of vertebral bodies were assessed. Density and dynamic histomorphometric measurements were made on cancellous bone. The results demonstrate that the structural biomechanical properties of vertebral bone are improved with the combination treatment. Stiffness and ultimate load of the OVX + RAL and OVX + ALN groups were significantly lower than those of sham animals, but the combination treatment with RAL + ALN was not significantly different from sham. Furthermore, the OVX + RAL + ALN group was the only agent-treated group in which the ultimate load was significantly higher than that in OVX animals (*p* < .05). Cancellous bone fractional volume (BV/TV_canc_) and bone mineral density (aBMD) also were improved with the combination treatment. BV/TV_canc_ of the OVX + RAL + ALN group was 6.7% and 8.7% greater than that of the OVX + RAL (*p* < .05) and OVX + ALN (*p* < .05) groups, respectively. Areal BMD of the OVX + RAL or OVX + ALN groups was not significantly different from that in OVX animals, but the value in animals undergoing combination treatment was significantly higher than that in OVX or OVX + RAL animals alone and not significantly different from that in sham-operated animals. Turnover rates of both the RAL + ALN and ALN alone groups were lower than in the RAL-treated alone group (*p* < .05). We conclude that the combination treatment of raloxifene and alendronate has beneficial effects on bone volume, resulting in improvement in the structural properties of vertebral bone. © 2011 American Society for Bone and Mineral Research.

## Introduction

Raloxifene (RAL), a selective estrogen receptor modulator (SERM), and alendronate (ALN), a bisphosphonate, reduce vertebral fracture risk by nearly the same extent despite variable effects on bone mineral density (BMD).(1–3) Compared with placebo, RAL treatment suppressed bone turnover and increased lumbar spine BMD by about half as much as ALN, but both agents produced similar reductions in vertebral fracture risk.(1–7) The contribution of the increase in BMD accounts for only 4% of the vertebral fracture reduction with RAL compared with 17% with ALN.(1,3,6,7) These data suggest that RAL and ALN improve the biomechanical properties of vertebral bone by different mechanisms.

Consistent with the clinical data, previous studies in an animal model have shown that the clinical dose of RAL alters the properties of canine vertebral bone in ways that differ from bisphosphonates.(3,8–11) ALN has negative effects on the derived material properties (structural properties normalized by bone geometry and fractional bone volume), but these negative effects are counteracted by an increase in bone volume such that there is no deterioration of the biomechanical properties at the structural level.(11) On the other hand, RAL does not increase bone volume as much as ALN but improves the biomechanical properties of bone by having positive effects on the derived material properties.(9) Since treatment with ALN is extending beyond a decade in some patients, the potential negative effects of ALN on the material properties could override the beneficial effects of the increase in bone volume, leading to an impairment of the biomechanical properties at the structural level. Treatment with both RAL and ALN (RAL + ALN) could offset part or all of the deterioration in bone's material properties that may be associated with ALN and concomitantly increase BMD.

Johnell and colleagues(12) investigated the additive effects of RAL and ALN on BMD and biochemical markers of bone turnover in postmenopausal women with osteoporosis. They found that treatment with both RAL and ALN results in a greater BMD increment at the femoral neck than did monotherapy with either agent.(12) Although BMD at the lumbar spine of the patients who were treated with RAL + ALN was different only from those treated with RAL alone, the authors concluded that the effects of RAL and ALN on BMD are independent and additive.(12) However, the effects of RAL + ALN on the structural and material biomechanical properties of bone were not determined in that study.

The goal of this study was to investigate the additive effects of RAL and ALN on vertebral bone in an estrogen-deficient animal model. We hypothesized that the combination of RAL and ALN will improve bone's structural properties more than each agent alone by allowing the ALN-induced increase in bone volume but preventing the negative effects of this bisphosphonate on bone's derived material properties by cotreatment with RAL.

## Materials and Methods

### Animals

Eighty-six-month-old Sprague-Dawley rats were obtained from Harlan Laboratories (Indianapolis, IN, USA) and randomized into five experimental groups (sham, OVX, OVX + RAL, OVX + ALN, and OVX + RAL + ALN; *n* = 16/group). All rats except those in the sham-operated group were subjected to bilateral ovariectomy. Compound administration was initiated after an acclimation period of 17 days following ovariectomy. RAL (0.5 mg/kg/day), ALN (1.0 µg/kg/day), RAL (0.5 mg/kg/day) + ALN (1.0 µg/kg/day), or daily saline vehicle (in equivalent volume to the drug treatments) were given subcutaneously. The doses of RAL (0.5 mg/kg/day) and ALN (1.0 µg/kg/day) approximate the clinical treatment dose for postmenopausal women.(3,13,14) ALN was purchased from Sigma-Aldrich (St Louis, MO, USA) and RAL was provided by Eli Lilly and Co. (Indianapolis, IN, USA). All animals were pair housed under standard laboratory conditions and had free access to food (2014 Teklad Global 14% Protein Rodent Maintenance Diet, Indianapolis, IN, USA) and water. One rat in the OVX + ALN group was removed from the study owing to illness. Animals were euthanized 16 weeks after the initiation of treatment. All rats were double-labeled with calcein (10 mg/kg of body weight, i.p.) with a 7-day interlabel period and a 3-day period for washout (ie, 1-7-1-3). Following euthanization, the lumbar vertebrae and tibias were collected and stored. All procedures were approved by the Indiana University School of Medicine Animal Care and Use Committee.

### Densitometry

Whole L_6_ vertebrae were scanned by micro–computed tomography (µCT; µCT40, Scanco Medical, Bassersdorf, Switzerland) to determine vertebral cross-sectional area, bone fractional volume, and trabecular microarchitecture. Prior to scanning, the posterior elements and transverse processes were removed by a bone cutter. The end plates also were removed using a low-speed diamond saw (Isomet 1000 Precision Saw, Beuhler, Lake Bluff, IL, USA). Removal of the cranial/caudal endplates was done such that the bone surfaces were parallel for mechanical testing.(15) Four L_6_ vertebrae (sham, *n* = 1; ALN, *n* = 2; OVX + RAL + ALN, *n* = 1) were substituted with the corresponding L_5_ vertebrae because they fractured during the cutting process. The specimens were imaged at 12-µm resolution, 55-kVp voltage, and 145 µA. A representative vertebral cross-sectional area (CSA, mm^2^) value for each vertebra was calculated as the average of the CSA measured at three different locations (25%, 50%, and 75% of total vertebral height).(15) Bone fractional volume and trabecular microarchitecture were analyzed (σ = 0.8, support = 2) in a 1.2-mm region directly above the caudal growth plate. This region was selected to avoid any errors introduced by the anterior venous foramen. The following parameters were obtained: whole vertebra (cancellous and cortical bone) fractional volume (BV/TV_whole vert_), cancellous bone fractional volume (BV/TV_canc_), trabecular thickness (Tb.Th, µm), trabecular number (Tb.N, mm^−1^), trabecular separation (Tb.Sp, µm), and structural model index (SMI). For the cancellous bone parameter (BV/TV_canc_, Tb.Th, Tb.N, Tb.Sp, and SMI) analysis, the cortical shell was removed using an adapted segmentation algorithm “dual threshold”.(16)

Following µCT scans, areal bone mineral density (aBMD, g/cm^2^) of the lumbar vertebrae was assessed using a PIXImus II densitometer (Lunar Corp., Madison, WI, USA). The specimens were scanned in the anteroposterior direction (ie, the posterior side was placed down).(17)

### Biomechanical testing

Biomechanical properties were obtained on the same vertebrae that were analyzed for densitometry. Testing was performed under uniaxial compression loading (0.5 mm/min) on a servo hydraulic testing machine (858 Mini Bionix II, MTS Corp., Eden Prairie, MN, USA).(17) Load-displacement data were recorded at a frequency of 100 Hz. The specimens were glued to the compression platens during testing. Structural (extrinsic) properties included stiffness (slope of the linear portion of load-displacement curve *S*, N/mm), ultimate load (maximum load obtained during testing *UL*, N), and work to failure (area under the load-displacement curve up to the ultimate load *W*, mJ). Derived material (intrinsic) properties (normalized stiffness *nS*, normalized ultimate load *nUL*, normalized work to failure *nW*) were calculated by normalizing the structural properties to bone geometry and whole vertebra fractional volume using the following equations(15,18):


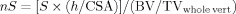



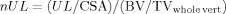



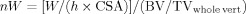


where *h* is the specimen height, measured using digital calipers prior to mechanical testing.

### Histomorphometry

Histologic measures were obtained on cancellous bone of the right proximal tibia to assess the effectiveness of the drug treatments. The right tibias were placed in 10% phosphate-buffered formalin for 3 days and then transferred to 70% ethanol until processing.(15,18) The specimens were dehydrated through a graded series of ethanols (70% to 100%) using an automatic tissue processor (Shandon/Lipshaw, Pittsburgh, PA, USA).(15,18) Following dehydration, the tibias were cleared with xylenol and embedded in methyl methacrylate (MMA; Sigma Aldrich, St Louis, MO, USA), as described previously.(15) Transverse sections from the proximal tibia were cut at 4 µm thick using a microtome (Leica RM2253, Richmond, IL, USA) and left unstained for dynamic histomorphometry measures. The sections were mounted on glass slides using Eukitt's glue (Electron Microscopy Sciences, Hatfield, PA, USA).

Dynamic histomorphometric measurements were performed via a semiautomatic analysis system (Bioquant OSTEO 7.20.10, Bioquant Image Analysis Co., Nashville, TN, USA) connected to an epifluorescence microscope (Nikon Optiphot 2 Microscope, Nikon, Melville, NY, USA).(15) A sampling region of approximately 8 mm^2^ was examined from the right proximal tibia. The measurements were done in the secondary spongiosa, 1 mm distal from the end plate. Dynamic histomorphometric parameters included mineralizing surface (MS/BS), mineral apposition rate (MAR, µm/day), and bone formation rate (BFR/BS, µm^3^/µm^2^/year). Two specimens in the OVX + RAL + ALN group and one specimen in the OVX + RAL group did not have double label and were assigned a value of 0.3 µm/day for MAR.(19,20) Dynamic variables were measured and calculated in accordance with ASBMR recommended standards.(21)

### Statistics

The differences among the groups (sham, OVX, OVX + RAL, OVX + ALN, and OVX + RAL + ALN) were examined using one-way analysis of variance (ANOVA) tests following Anderson-Darling normality tests. When a significant overall *F* value was present (*p* < .05), Fisher's protected least-significant-difference (PLSD) post hoc tests were used to compare differences between individual group means. For variables violating the normality assumption, Kruskal-Wallis tests were used. When the Kruskal-Wallis test revealed a significant difference (*p* < .05), it was followed by Mann-Whitney pairwise comparisons between individual group medians. For all tests, *p* < .05 was considered statistically significant. MINITAB 15 software (Minitab, Inc., State College, PA, USA) was used for all the statistical analyses.

## Results

**Fig. 1:**
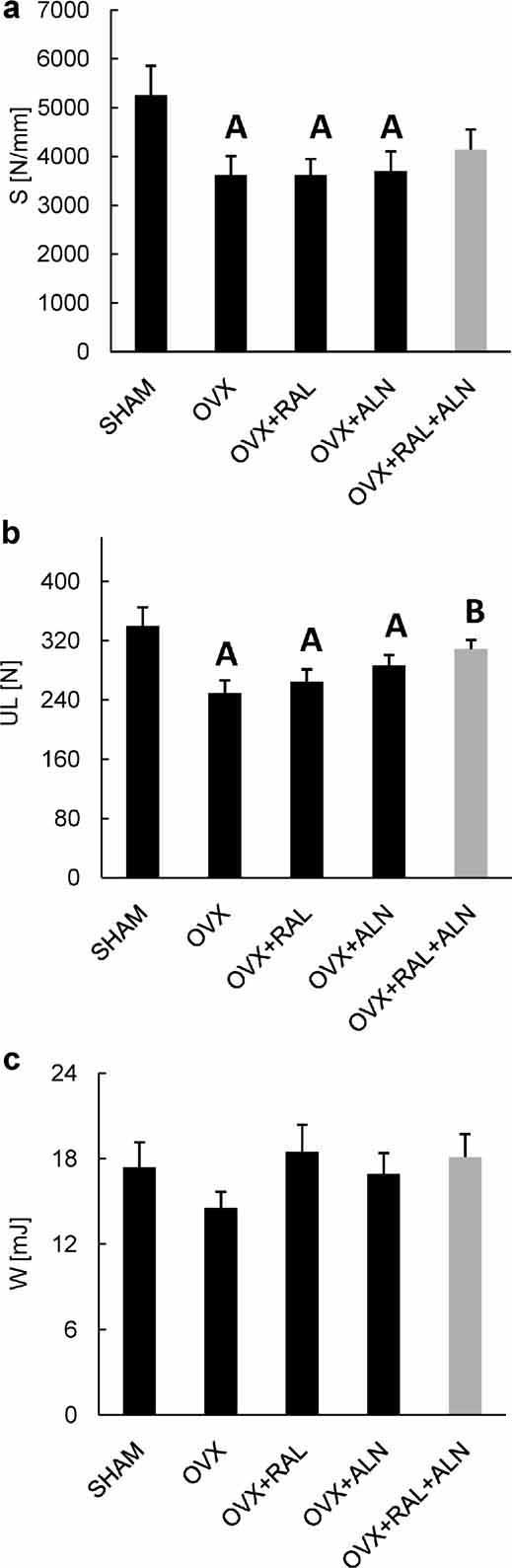
Structural biomechanical properties of the vertebral body following treatment: (*a*) Stiffness *S* (*p*_ANOVA_ = .043); (*b*) ultimate load *UL* (*p*_ANOVA_ = .004); (*c*) Work to failure *W* (*p*_Kruskal-Wallis_ = .686). (*A*) Significantly different from sham; (*B*) significantly different from OVX. Data are presented as ± SE mean.

The combined treatment of RAL and ALN had beneficial effects on the structural biomechanical properties of vertebral bone. Stiffness and ultimate load of the OVX + RAL + ALN group were not significantly different than those of the sham-operated group (Fig. 1*A, B*). In contrast, when either agent was administered alone (OVX + RAL or OVX + ALN) stiffness and ultimate load were significantly lower than sham (Fig. 1*A, B*; *p* < .05). Furthermore, the OVX + RAL + ALN group was the only agent-treated group in which the ultimate load was significantly higher (+23.8%) than that of OVX (Fig. 1*B*; *p* < .05). No differences in the work to failure (Fig. 1*C*) or in any of the derived material biomechanical properties were found (Table 1).

**Table 1:** Derived Material Properties of Vertebral Bone Following Treatment

	Sham	OVX	OVX + RAL	OVX + ALN	OVX + RAL + ALN	*p* Value
*nS* (GPa)	5.69 ± 0.59	5.61 ± 0.57	4.69 ± 0.43	5.02 ± 0.58	4.92 ± 0.48	*p*_Kruskal-Wallis_ = .633
*nUL* (MPa)	69.06 ± 4.39	68.21 ± 3.96	61.79 ± 4.11	68.09 ± 3.45	67.08 ± 2.24	*p*_Kruskal-Wallis_ = .611
*nW* (MPa)	0.67 ± 0.07	0.69 ± 0.04	0.79 ± 0.08	0.71 ± 0.08	0.74 ± 0.08	*p*_Kruskal-Wallis_ = .599

Data are presented as ± SE mean. *nS* = normalized stiffness; *nUL* = normalized ultimate load; *nW* = normalizd work to fracture.

**Fig. 2:**
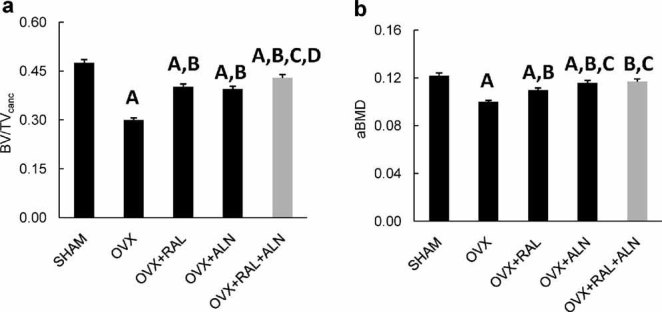
Bone volume measurements following treatment: (*a*) Cancellous bone fractional volume (BV/TV_canc_) (*p*_ANOVA_ < .001); (*b*) aBMD of the vertebral body (*p*_ANOVA_ < .001). (*A*) Significantly different from sham; (*B*) significantly different from OVX; (*C*) significantly different from OVX + RAL; (*D*) significantly different from OVX + ALN. Data are presented as ± SE mean.

The differences in structural properties were associated with changes in fractional bone volume and bone mineral density. BV/TV_canc_ of the RAL + ALN group was 6.7% and 8.7% higher than that of the RAL (*p* < .05) and ALN (*p* < .05) groups, respectively (Fig. 2*A*). Unlike both the OVX + ALN and OVX + RAL groups, which had significantly lower aBMD than sham (Fig. 2*B*; *p* < .05), the OVX + RAL + ALN group aBMD was not significantly different from sham (Fig. 2*B*). The OVX + RAL + ALN group had a 6.3% higher aBMD than the OVX + RAL group (Fig. 2*B*; *p* < .05).

The combined treatment also resulted in positive effects on cancellous bone microarchitecture. Tb.N and Tb.Sp of the combination treatment group were not significantly different from those of the sham group (Table 2). On the other hand, both the OVX + RAL and the OVX + ALN groups had a lower Tb.N and greater Tb.Sp than sham (Table 2; *p* < .05). The RAL + ALN treatment significantly increased Tb.N (Table 2; +7%, *p* < .05) and decreased Tb.Sp (Table 2; −8.6%, *p* < .05) relative to treatment with RAL alone. In addition, SMI of the OVX + RAL + ALN group was 40.3% lower (more negative) than that of the OVX + ALN group (Table 2; *p* < .05), suggesting that the trabecular plates of the OVX + RAL + ALN group contain more closed cavities between trabeculae.(22) No beneficial effect in Tb.Th with the combination treatment compared with the monotherapy treatments was detected.

**Table 2:** Cancellous Bone Microarchitecture Parameters Following Treatment

	Sham	OVX	OVX + RAL	OVX + ALN	OVX + RAL + ALN	*p* Value
Tb.Th. (µm)	90.39 ± 1.20	74.23 ± 0.90^a^	82.79 ± 1.12^a^ ^b^	80.28 ± 1.33^a^ ^b^	82.83 ± 1.33^a^ ^b^	*p*_ANOVA_ < .001
Tb.N. (mm^−1^)	5.50 ± 0.07	4.31 ± 0.07^a^	4.97 ± 0.09^a^ ^b^	5.16 ± 0.04^a^ ^b^	5.32 ± 0.10^b^ ^c^	*p*_ANOVA_ < .001
Tb.Sp. (µm)	162.74 ± 3.07	223.08 ± 3.76^a^	186.38 ± 4.39^a^ ^b^	177.85 ± 2.23^a^ ^b^	170.39 ± 3.86^b^ ^c^	*p*_Kruskal-Wallis_ < .001
SMI	−2.31 ± 0.16	−0.22 ± 0.06^a^	−1.41 ± 0.09^a^ ^b^	−1.19 ± 0.09^a^ ^b^	−1.67 ± 0.14^a^ ^b^ ^d^	*p*_ANOVA_ < .001

Data are presented as ± SE mean. Tb.Th = trabecular thickness; Tb.N = trabecular number; Tb.Sp = trabecular separation; SMI = structural model index.

a Significantly different from sham.

b Significantly different from OVX.

c Significantly different from OVX + RAL.

d Significantly different from OVX + ALN.

Dynamic histomorphometric measures revealed differences when the two agents were combined. Bone formation rate (surface-based remodeling rate) of the OVX + RAL + ALN group was 68.5% lower than that of the OVX + RAL group (*p* < .05; Table 3). The difference in bone turnover rate between the OVX + RAL + ALN and OVX + RAL groups was achieved mainly by a lower MS/BS (Table 3; −65.1%, *p* < .05), which was suppressed about 25% more than with ALN alone.

**Table 3:** Cancellous Bone Dynamic Histomorphometric Parameters Following Treatment

	Sham	OVX	OVX + RAL	OVX + ALN	OVX + RAL + ALN	*p* Value
MS/BS %	8.86 ± 0.82	19.15 ± 1.52^a^	13.11 ± 1.36^a^ ^b^	5.96 ± 1.16^a^ ^b^ ^c^	4.57 ± 0.33^a^ ^b^ ^c^	*p*_Kruskal-Wallis_ < .001
MAR (µm/day)	1.12 ± 0.04	1.03 ± 0.04	1.00 ± 0.07	0.77 ± 0.04^a^ ^b^ ^c^	0.85 ± 0.09^a^ ^b^	*p*_ANOVA_ < .001
BFR/BS (µm^3^/µm^2^/year)	36.0 ± 3.4	71.9 ± 5.9^a^	46.3 ± 4.7^b^	18.0 ± 0.7^a^ ^b^ ^c^	14.6 ± 1.9^a^ ^b^ ^c^	*p*_Kruskal-Wallis_ < .001

Data are presented as ± SE mean. MS/BS % = mineralizing surface; MAR = mineral apposition rate; BFR/BS = bone formation rate.

a Significantly different from sham.

b Significantly different from OVX.

c Significantly different from OVX + RAL.

## Discussion

Following 16 weeks of dosing, the combination of RAL and ALN increased bone volume more than either agent administered alone, resulting in an improvement in the extrinsic biomechanical properties. The results suggest that for osteoporotic patients who are at a higher fracture risk owing to elevated bone loss, the combined treatment of RAL and ALN may be more efficacious than treatment with RAL or ALN alone. To our knowledge, this is the first study to report the effects of combining these two different treatment regimens on bone volume and biomechanical properties.

In a canine model, it has been shown that ALN and RAL improve the biomechanical properties of bone by different mechanisms. Compared with vehicle-treated animals, treatment with ALN had negative effects on the derived material properties, but these negative effects were counteracted by an increase in bone volume such that there was no deterioration to bone's structural properties.(11) On the other hand, treatment with RAL improved the derived material properties compared with vehicle-treated animals but did not increase bone volume as much as ALN.(9) Consistent with these data, Allen and colleagues(3) have found that compared with treatment with ALN, treatment with RAL resulted in an improvement in the derived material properties. We hypothesized, therefore, that the combination of RAL and ALN will improve bone's structural properties more than each agent alone by allowing the ALN-induced increase in bone volume and preventing the negative effects of ALN on bone's derived material properties by cotreatment with RAL. The results reported here show that compared with the OVX group, the treatment with RAL or ALN alone did not result in significant changes in the derived material properties (Table 1). Thus the additive positive effects of the interaction between RAL and ALN on the structural properties of vertebral bone found here are not because the cotreatment with RAL had prevented the negative effects of ALN on the derived material properties. One possible explanation for the differences in the derived material properties between the canine study and this investigation could be related to the treatment duration. In the study of Allen and colleagues,(3) the dogs were treated for 1 year, whereas the rats in our study were treated for 16 weeks. Although the number of remodeling cycles during the treatment duration in these two studies is almost the same,(23–25) the mean age of the bone matrix following treatment will be different in these animal models. This is so because the mean age of the bone matrix is a function of both the treatment duration and the number of remodeling cycles during the treatment. The mean age of the bone matrix is deemed to be associated with changes in the tissue matrix properties (ie, bone microstructure and ultrastructure), which, in turn, have a direct effect on bone's material properties.(8,26,27) Since rodents have a short life span compared with larger animals, future studies investigating the long term additive effects of RAL and ALN on the material properties of bone should be done in a large animal model.

In this study, we found significant declines in vertebral microarchitecture and a significant increase in BFR/BS in OVX animals compared with the sham group, showing the effectiveness of the ovariectomy. However, we did not find significant differences in any of the derived material properties between the sham and OVX groups. Although this could be a function of the age of the animals and the duration of treatment, a more likely explanation is that ovariectomy alters structural properties of vertebral trabecular bone but does not significantly change the properties of the bone tissue itself.

The results of this investigation are consistent with those of Johnell and colleagues,(12) who showed that in postmenopausal women with osteoporosis, the effects of RAL and ALN on BMD are independent and additive when given in combination. RAL + ALN produced a greater incremental increase in BMD at the femoral neck than each agent alone.(12) The lumbar spine BMD of the RAL + ALN group was roughly the same as in the ALN alone group but was significantly higher than in the RAL alone group.(12) They also reported that patients who received RAL + ALN or ALN alone treatment had similar levels of bone turnover but lower levels than those who received RAL.(12) We demonstrated that RAL + ALN treatment resulted in a higher cancellous fractional bone volume than monotherapy with either RAL or ALN alone. The changes in the lumbar vertebral aBMD reported here were similar to the changes in lumbar spine BMD reported by Johnell and colleagues(12) (Fig. 2*B*). Furthermore, we found that the turnover rate of the animals treated with RAL + ALN reached significance only when compared with those treated with RAL alone (Table 3).

It is noteworthy that although aBMD is often used as a clinical surrogate for bone strength, it is a poor indicator of bone density.(1,6,7) This is so because aBMD provides a low-resolution 2D projection of bone mineral content and cannot differentiate whether the changes occur in cancellous or cortical bone.(28,29) In contrast, BV/TV_canc_, as measured in this investigation, is a high-resolution 3D assessment of bone density and is specific to cancellous bone.(28,29) The effects of antiremodeling agents on bone density are more pronounced in cancellous bone.(20) A high-resolution imaging modality is needed to detect such effects. BV/TV_canc_ therefore is a better marker than aBMD in evaluating the efficacy of the combination treatment.

The combined use of RAL + ALN also led to an improvement in trabecular number and separation more than could be achieved with either agent alone (Table 2). SMI of the OVX + RAL + ALN group was more negative than that of the ALN group. As described by Mastbergen and colleagues,(22) a more negative SMI suggests that the trabecular plates contain more closed cavities between trabeculae.(22) The conversion from open to closed trabecular plates is related to an increase in bone volume and trabecular thickness.(22) Taken together, the addition of RAL to an ALN regimen may enhance bone microarchitecture, one measure of bone quality, substantially in osteoporotic patients.

This study has various limitations. First, the derived material properties reported here are only an estimate of the true material properties. This is so because it is not possible to fully take into account bone geometry, fractional volume, and microarchitecture in calculating the derived material parameters. Second, four L_6_ vertebrae were substituted with the corresponding L_5_ vertebrae because they fractured during the cutting process. However, all the experimental values from each substituted vertebra were within 3 SD of the mean values for L_5_ vertebrae within their respective groups. Third, the histomorphometric analysis of cancellous bone was done on the proximal tibia, whereas the biomechanical properties were obtained from the vertebra. The goal of calculating the histomorphometric parameters was to assess the effect of RAL + ALN treatment on the turnover rate of cancellous bone. Because the effects of ovariectomy occur earlier in the proximal tibia than vertebra,(30,31) we chose to conduct the histomorphometric analysis on the proximal tibia. We expect, though, that the effects of RAL + ALN on turnover rate will be similar among different cancellous bone sites. Furthermore, the changes in bone turnover rate found in this investigation are in good agreement with clinical data.(12)

In conclusion, in an ovariectomized adult rat model, which is an established model for estrogen-deficiency osteoporosis, we showed that the combination of RAL and ALN has greater beneficial effects on bone volume and biomechanical properties of vertebral bone compared with either agent alone. The findings of this work provide new insight into the effects of combining two different osteoporosis treatment modalities on bone fragility. Further studies should be done in large animal models to ascertain the positive effects of the combination treatment of RAL and ALN on vertebral bone observed here.
